# Morphine aggravates inflammatory, behavioral, and hippocampal structural deficits in septic rats

**DOI:** 10.1038/s41598-023-46427-y

**Published:** 2023-12-05

**Authors:** Evans O. Ayieng’a, Elham A. Afify, Salwa A. Abuiessa, Samar S. Elblehi, Sahar M. El-Gowilly, Mahmoud M. El-Mas

**Affiliations:** 1 Department of Pharmacology and Toxicology, Faculty of Pharmacy, Alexandria University, 1-El-Khartoum Square-Azarita, Alexandria, 21521 Egypt; 2 Department of Pathology, Faculty of Veterinary Medicine, Alexandria University, Alexandria, Egypt; 3 Department of Pharmacology and Toxicology, College of Medicine, Kuwait University, Kuwait, Kuwait

**Keywords:** Neuroscience, Neurology

## Abstract

Although pain and sepsis are comorbidities of intensive care units, reported data on whether pain control by opioid analgesics could alter inflammatory and end-organ damage caused by sepsis remain inconclusive. Here, we tested the hypothesis that morphine, the gold standard narcotic analgesic, modifies behavioral and hippocampal structural defects induced by sepsis in male rats. Sepsis was induced with cecal ligation and puncture (CLP) and behavioral studies were undertaken 24 h later in septic and/or morphine-treated animals. The induction of sepsis or exposure to morphine (7 mg/kg) elicited similar: (i) falls in systolic blood pressure, (ii) alterations in spatial memory and learning tested by the Morris water maze, and (iii) depression of exploratory behavior measured by the new object recognition test. These hemodynamic and cognitive defects were significantly exaggerated in septic rats treated with morphine compared with individual interventions. Similar patterns of amplified inflammatory (IL-1β) and histopathological signs of hippocampal damage were noted in morphine-treated septic rats. Additionally, the presence of intact opioid receptors is mandatory for the induction of behavioral and hemodynamic effects of morphine because no such effects were observed when the receptors were blocked by naloxone. That said, our findings suggest that morphine provokes sepsis manifestations of inflammation and interrelated hemodynamic, behavioral, and hippocampal deficits.

## Introduction

Sepsis, a fatal multiorgan failure syndrome attributed to deranged host infection-response and generalized inflammation^1^. Over 45 million people suffer from sepsis annually with at least 1 death in every 5 patients^2^ which reflects a major health problem. The percentage of deaths is reported to be doubled in septic patients admitted to intensive care unit (ICU)^3^. Individuals who survive sepsis are often burdened with cognitive impairment and neurological complications^4^. Experimentally, sepsis is found to be associated with dysregulated cardiovascular^5,6^, behavioral^7^ and cognitive profiles^8^. Sepsis-associated encephalopathy, a diffuse brain dysfunction secondary to infections outside the central nervous system, develops in approximately 70% of septic patients^4^. The cytokine storm incites central neuroinflammatory pathways within the hypothalamic pituitary axis, hippocampus, and amygdala, thereby interrupting memory consolidation^9^. Sepsis increases the permeability of the blood brain barrier (BBB) to circulating cytokines within 24 h of cecal ligation and puncture (CLP)^10^. BBB integrates signals from the brain and the periphery in sepsis such as neuroinflammation, increased barrier permeability and immune cell infiltration^11^ The infection triggers the host immune response leading to vascular endothelial damage, interruption of tight junctions’ proteins; loss of integrity of BBB, allowing the entry of peripheral immune cells into the brain, which triggers the activation of glial cells and neuroinflammation^12^. Experimental evidence suggest that, during systemic infection, elevated levels of inflammatory cytokines alter synaptic GABAergic transmission^13^ and contributes to cognitive dysfunction observed in sepsis^14^. Furthermore, oxidative damage caused by sepsis increases lipid peroxidation in prefrontal cortex, hippocampus, and striatum, which contributes to long-lasting cognitive and functional impairment associated with sepsis in rats^15^.

Like sepsis, behavioral functions have been shown to be adversely influenced by morphine and other narcotic analgesics. For instance, experimental data showed that morphine enhances the production of inflammatory and apoptotic molecules that increase the expression of amyloid precursor protein and amyloid genesis and cause cognitive impairment^16,17^. Animal studies demonstrated that morphine attenuates the learning behaviour^18^, memory consolidation^19^, swimming speed, and spatial memory^20^. Evidence obtained from pharmacologic antagonist studies suggests a pivotal role for opioid receptors in behavioral anomalies elicited by morphine^21–23^.

Despite the apparently similar inflammatory and behavioral consequences of sepsis^24,25^ and narcotic analgesics^26–28^ when applied individually, the question whether simultaneous exposure to the two challenges would provoke a more deteriorated behavioral response remains elusive. The validity of this postulate gains support from the observation that morphine as such may induce sepsis and septic shock via tempering endotoxin tolerance and inducing persistent inflammation^29–32^. Morphine activates toll-like receptor 4 (TLR4) and upregulates proinflammatory cytokine production. It also permits the leakage of Gram-negative bacteria and lipopolysaccharides (LPS) from the intestinal lumen. Further, morphine impairs mucosal barrier integrity via TLR4-mediated negative crosstalk pathways, which fosters translocation of gut microflora, increasing the risk of infection^29^. Clinically, Glattard et al.^33^ reported dramatically increased levels of endogenous morphine in the serum of patients with generalized infection in sepsis. Experimental LPS challenge of morphine treated animals accentuates proinflammatory cytokines TNF-α, IL-1β, and IL-6, and reduces serum corticosterone, which act synergistically to enhance the development of sepsis and septic shock^34^. Recently Abu et al.^35^ concluded that, clinical and preclinical data advocate that opioids increase sepsis risk possibly via microbiome and immune modulation with prominent involvement of miR-146a, which plays key roles in endotoxic tolerance. Both clinical^36^ and experimental^37^ data have shown that opioid-treated septic subjects demonstrate significantly higher mortality rates. Here, studies were undertaken to test the hypothesis that morphine exacerbates cardiovascular and behavioral induced by sepsis in rats. Histopathological studies were also pursued to identify the role of structural defects in the hippocampus, which is tightly linked to memory and cognition control, in the evoked responses. All measurements were taken 24 h following isolated or combined exposure of rats to the septic and morphine interventions.

## Materials and methods

### Animals

Adult male Wistar rats (170 to 240 g) were procured from the Faculty of Pharmacy Animal Facility, Alexandria University, Alexandria, Egypt. Rats were housed in plastic boxes in groups of six with alternate 12 h light and dark cycles. Food was available ad libitum. All methods were performed in accordance with the relevant guidelines and regulations**.** All methods were performed in accordance with the relevant guidelines and regulations of ARRIVE guidelines (https://arriveguidelines.org) and were approved by the Alexandria University Animal Care and Use Committee (approval No. MS (3) 17). First, all rats were tested for general motor activity in open field test. Animals with very low activities are excluded from the beginning and animals with high motor activity (horizontal and vertical activities) are included.

### Protocols and experimental groups

The present study adopted a previously conducted design^38^ where four behavioral tests were carried out. Most tests can be conducted 24 h after CLP to determine the associated cognitive impairments^39,40^. In the current study, tests were carried out sequentially 24 h after CLP on the same day and videotaped. Blood pressure was measured followed by Y-maze test, New object recognition test (NORT) and Morris water maze (MWM).

CLP or sham-operated male rats (2 groups each, n = 6–9 per group) were allocated to receive two s.c. injections of saline 0.9% or morphine (7 mg/kg) 24 and 6 h prior to sham or CLP. To determine the role of opioid receptors in the morphine responses, another two sham groups were employed that received 2 doses of naloxone (µ-opioid receptor blocker, 0.5 mg/kg s.c.) or naloxone plus morphine. The naloxone doses were administered 30 min before respective morphine doses. Twenty-four hours after CLP or sham procedures, all groups were processed for the assessment of hemodynamic (SBP by tail-cuff plethysmography) and behavioral profiles (tail flick, Y-maze, Morris Water Maze and new object recognition). Rats were anesthetized with thiopental (50 mg/kg i.p), blood samples were collected from the retroorbital sinus, centrifuged, and serum was aspirated and stored at − 80 °C for biochemical determinations of the inflammatory cytokine IL-1ß. Rats were finally euthanized by an overdose of thiopental (100 mg/kg i.p.), brains were removed, and hippocampal tissues were excised and processed for histopathological studies.

### Induction of sepsis

The CLP procedure was used to induce sepsis as previously described^41,42^. Rats were anaesthetized with thiopental (50 mg/kg, ip), fixed in the supine position and a 1 cm midline abdominal incision was made. The underlying muscles were exposed, and a 1-cm midline was made on the ventricular surface of the abdomen, then the cecum was exposed and ligated 1cm away from the base. Two punctures were made using a 21-gauge needle and fecal matter was expressed. A group of male rats underwent surgical procedures only (sham). The cecum was exposed under anaesthesia and left for 2 min after laparotomy and returned to the abdominal cavity without ligation and puncture. The abdominal muscles and skin were closed with simple running sutures and animals were allowed 24 h before processing the behavioral and biochemical tests.

### Tail flick test

The tail flick method was employed to assess antinociception using an Algesimeter in which radiant heat was focused 15–22 mm from the tip of the tail^43^. Rats were tested three times on three separate days, and the average latency time was recorded as the baseline. The cut-off time was 10 s to avoid tissue amage. Rats were tested one h post administration of the tested drug or saline. Tail-flick latency data were expressed as maximum possible effect (MPE)^44^ where,

$$MPE\left(\%\right)=\frac{((Post{\text{-}}treatment\, latency)-(Pre{\text{-}}treatment\, latency))}{((Cut{\text{-}}off\, time)-(Pre{\text{-}}treatment \,latency))}\times 100.$$

### Behavioral tests

All animals were observed after CLP and/or morphine injection to determine signs of lethargy and to exclude animals showing signs of disability and distress. After CLP or morphine injection, rats showing shallow and rapid respiration, piloerection, hunching or cowering in the corner of the cage, belly-pressing, twitching, or staggering or defective walking or running during training periods in all the mazes were excluded^45^. A few numbers of rats were excluded throughout the study. Morphine alone was not associated with mortality during our study, but septic rats and morphine treated septic rats were associated with some mortality. All included rats showed normal behaviour post CLP or sham surgery. Behavioral procedures were conducted between 13:00 and 16:00 h in a sound-isolated room, tracked using video camera and analysed.

#### Y-maze test

A wooden 35 cm long, 12 cm width and 28 cm high Y-maze was tailored with three identical arms 120° angles apart. Arms were labelled A B C for ease of data interpretation^46^. Visual cues were placed on the edges of the maze. After habituation, an inter-trial interval (ITI) of 24 h was adopted and the test was carried out 24 h after the last training session. During the test session a hidden camera was placed, and the animals were left to explore the three arms freely for 5 min^47^.

##### Spontaneous alternation behaviour

The animals were gently held by the base of the tail and placed on the distal section of arm A, facing the center of the maze. Then each rat was given 5 min to explore the maze before the video was stopped, and the animal was retrieved and returned to its home cage. The number of all arm entries and alternations was counted. The percentage alternations were calculated as follows^48^ where higher % alternation indicates a better spatial working memory

$$\mathrm{\%Alternations }=\frac{number\, of \,alterations}{total\, number \,of\, arm\, entries-2} \times 100.$$

##### Time spent on novel arm

To test for the novel arm entry, the animals were placed in the maze facing the centre and left to explore with the novel arm closed for 10 min and then removed from the maze. The test animal is placed back into the maze for additional 5 min after removing the blockage of the novel arm. The time spent in the novel arm is calculated in seconds as previously reported^49^. Subsequently, the animals were returned to their home and left undisturbed for one hour before performing the Morris water maze test. All animal behaviors were tracked using a video camera.

Y maze test was performed by placing the animal in a maze for 5 min during the test period. 6–9 animals would take 30–45 min to complete the test. Additional 6–9 min are required for animal handling according to the number of rats in experimental group. Therefore, an approximate of 36–54 min are required to perform the experiment. Start of experiment 13:00 h- End of experiment 13:36–13:54 h.

#### New object recognition (NOR) test

The NOR test was used to determine the recognition memory of the animals. The test was carried out in a square open field made of plexiglass with dimensions 46 cm × 46 cm × 40 cm^50^. The floor of the open field was divided into 16 equal squares by black lines. The objects used were placed symmetrically 5 cm from the wall. All objects were thoroughly cleaned with 70% ethyl alcohol to avoid any olfactory cues. Objects were balanced to avoid any potential bias^51^. Twenty-four hours after habituation two similar objects were placed in opposite quadrants (i.e., NE corner and SW corner) of the box^50^. Rats were allowed to explore and familiarize with the objects for 10 min. One hour later, one of the familiar objects was replaced with a novel object and rats were left to explore it for 10 min. The activity during the test session was recorded using a hidden camera. The total exploration time during training for two identical objects was calculated by determining the total time the animal was able to explore the two identical objects. Similarly, the total exploration time during testing was the total time (10 min) that the animals explored the two items. The discrimination measure (DM) was calculated by determining the difference in time (seconds) between the time the animal actively explored the novel item from the familiar object. Animals were considered to have actively explored the items when they physically touched and climbed on the item and not moving besides the items. The discrimination index (DI) was calculated by dividing the discrimination measure with the total exploration time during testing. Preference index also known as the recognition index (RI) was the ratio obtained. For horizontal movements, each single horizontal movement of the animal is counted horizontally during test interval. The line crossing obtained by counting the total lines crossed by the animal and was evaluated as locomotor activity.

$$DM=time \,spent\, on \,novel\, object-time\, spent\, on\, familiar\, object,$$

$$DI=\frac{discrimination\, measure}{total\, exploration\, time} \qquad RI=\frac{time \,spent\, on\, novel\, object}{time\, spent \,on\, familiar \,object}.$$

**Figure 1:**
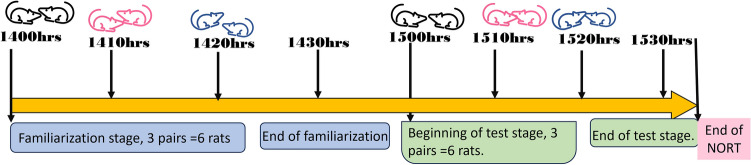
Time sequence of the new object recognition test.

To perform the NORT, two open fields were used and therefore two rats tested at the same time. A familiarization of 10 min and a test of 10 min with a 1-h break between familiarization and test was adopted in the time sequence presented in Fig. 1.

#### Morris water maze test (MWM)

Spatial learning and memory were assessed in a Morris water maze^52^. A plastic water tank with a diameter of 100 cm and a height of 50 cm was filled to a depth of 30 cm with water ± 20 °C. A hidden circular platform (with 15 cm diameter) made of Plexiglas was located in the northwest quadrant of the maze. For habituation, a training was conducted in clear water with the platform exposed 1 inch above the water level. Rats were placed gently in water and allowed to swim towards the platform. If the rats were unable to reach the platform in 60 s, they were guided. Rats were left on the platform for 20 s to enable them to remember the existence of the platform during the test. The trial was repeated for each animal in different quadrant^53^. This was done for 3 days before starting the experimental procedures. Distal and proximal cues were placed during the training session to help the rats determine the platform quadrant before the test^54^. All the quadrants were labelled N, S, E, and W and the animal was placed in all four quadrants to help navigate and find the platform^55^. A total of 12 training sessions were done per animal before the test.

##### Probe trial

After the training session, the animals were subjected to a probe trial in which the water was coloured with corn starch to make the platform invisible to the rats with the platform submerged 1 inch below the water. Each animal was given 2 min break in all trials before conducting another trial. The test was carried out by removing the hidden platform from water coloured with corn starch and the activity of the animal was recorded for 60 s^53^.

##### Determination of behavioral parameters in MWM

The distance travelled was estimated by calculating the total distance travelled in 60 s. The circumference of the MWW was calculated from the diameter (100 cm) and used to estimate the distances travelled by the animals along the wall of the cylinder. Time spent on the platform quadrant was estimated by calculating the total time the animals spent on quadrant southwest (SW) after the removal of the platform during the test. Escape latency was determined by estimating the time it took the animal to trace and stop at the platform during the probe trial. The number of crossings were counted when the animals crossed from any quadrant to the platform quadrant.

The total distance moved by rats is estimated by adding up distances measured from one point to another in a circle according to the distance formula:

$$d = \sqrt {\left( {x_{2} - x_{1} } \right)^{2} + \left( {y_{2} - y_{1} } \right)^{2} } ,$$

where *d* = distance; (*x*_1_, *y*_1_) = coordinates of the first point; (*x*_2_, *y*_2_) = coordinates of the second point.

**Figure 2:**
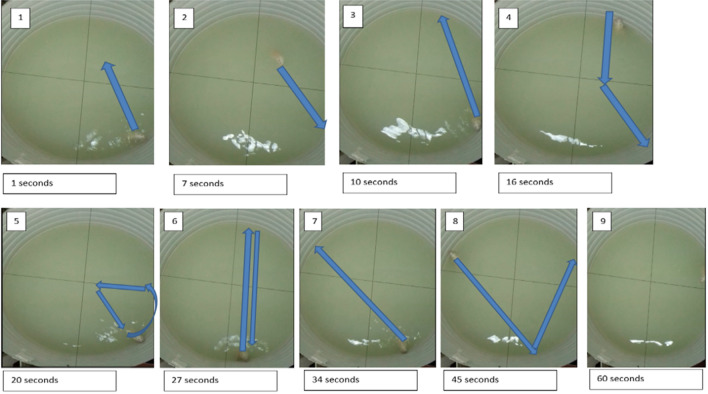
A Layout out of the procedure for calculating the distance travelled in Morris Water Maze.

This formula applies to all coordinates to estimate distance between points. Taking into consideration that the diameter of MWM is 100 cm, the radius is 50 cm and circumference C = 2πr which is 314.2 cm. The distance was measured accordingly. The diameter, radius and circumference were mapped from photos to estimate the distance between points. A Layout of distance moved and procedure for calculating the distance travelled are shown in the Fig. 2. The pictures are screen shots of the video in the experiment.

To calculate the number of crossings to the platform quadrant, the maze was divided virtually into 4 quadrants (northeast, northwest, southeast and southwest) by a north–south and east–west axis. All quadrants were divided using the strings tied on the top of the MWM. The arrows clearly mapped the movement of the rat with a crossing shown when the arrow intersected the string.

To perform this test. Each rat was tested for 60 s (1 min) in a probe trial (presence of submerged platform) and 2 min. Break before the next test without the platform. One group of rats (n = 6–9) would take 18–27 min to complete the experiment. Beginning or MWM = 15:30 h—End of experiment = 15:48–15:57 h.

Therefore, experiments were conducted between 13:00 h and 16:00 h for an average of between 6 and 9 rats. The number of animals could be varied according to the animal variations after statistical analysis.

### Histopathological examination

After euthanasia, the hippocampal specimens were isolated, rinsed in saline solution, and then placed for 24 h in 10% buffered formalin (pH 7.4). The processing of fixed specimens was done by the paraffin-embedding technique in accordance to Bancroft and Layton^56^. Sections of 4.5 µm thickness were obtained from the paraffin blocks using the Leica rotatory microtome and stained routinely with Mayer’s haematoxylin and eosin (H and E) and then the micrographs were obtained by digital camera (Leica EC3, Leica, Germany) linked to Leica DM500 microscope. The employed thickness was used by previous studies^57–60^. Briefly, digital images of H&E stained sections of the hippocampus (CA1) were uploaded into the Image J to quantify the number of different cell types. The CA1 region is the thickest layer of the hippocampus. It is composed of densely packed faint-stained pyramidal neurons. The degenerated neurons appear as shrunken cells with pyknotic and hyperchromatic nuclei. Microglia are small which exhibit elongated nuclei with little cytoplasm^59,61^.

### Histomorphometrical analysis

Quantitative analysis was performed using image J software (Image J v 1.46r, National Institute of Health, Bethesda, MD, USA) to quantify the number of different cell types in the hippocampal tissues of different rats’ groups (one section/animal; 5/group) at a magnification power of × 400^62^. All morphometric measurements were made blind.

### Tail-cuff plethysmography

Systolic blood pressure (SBP) of conscious rats was evaluated using a specific tail cuff and pulse transducer (Pan Lab, Spain) linked to a computerised data acquisition system with LabChart-7 pro software (Power Lab 4/30, model ML866/P, AD Instruments, Bella Vista, Australia)^63,64^. Rats were accustomed to enduring the warmed tail-cuff for at least 15 min for three consecutive days before the actual SBP measurement to avoid erroneous positive or negative BP responses. SBP was measured 3–4 times (5 min separated) at 8:00 am in the morning and values were averaged to the mean.

### Measurement of serum IL-1β concentration

Blood was withdrawn from the lateral saphenous vein of rats’ eye using a heparinized capillary tube into an ethylenediamine tetra-acetic acid (EDTA) tube to avoid coagulation 24 h post CLP/sham for determination of serum IL-1β concentration. Blood was immediately centrifuged at 5000 rpm for 5 min at room temperatures. The serum was aspirated and stored at − 80 °C until use. ELISA was used for the determination of IL-1β according to the manufacturer (Sigma Aldrich Co, USA).

### Drugs

Morphine (Masr Pharmaceutical Co., Cairo Egypt), Naloxone (SERB pharmaceutical Co., Paris France), Thiopental (Thiopental®, Biochemie GmbH, Vienna, Austria) and povidone-iodine solution (Betadine, Nile Pharmaceutical Co., Cairo, Egypt) were purchased from commercial vendors. All drugs were dissolved in saline.

### Statistical analysis

Values are expressed as means ± S.E.M. According to sample size calculation using G*power software version 3.1.9.7 with a statistical power of 80% and alpha 0.05 level of significance and effect size of 0.7, the number of animals per group was 7. Therefore, the range of animals employed in the study was n = 6–9 rats per group. The variation of number of animals among experimental groups related to discrepancies in experimental conditions such as animals’ variability in spatial aspects of movement, including distance, direction, area covered, and path tortuosity. This necessitated the use of a larger number of animals in certain groups as septic rats. Additionally, the limited availability of male rats and relatively higher mortality in septic groups were other reasons for the variability in sample size. One-way ANOVA followed by the Tukey’s post hoc test was used to measure statistical significance. Statistical analysis was performed using the GraphPad Prism software (Version 8.02). The Pearson correlation analysis was used to determine the linear relationship between behavioral and blood pressure responses in various experimental settings. The Pearson's correlation coefficient “r” measures the strength and direction of association that exists between the two presented variables. The line of best fit through the data of two variables, and the “r” value indicates how far away data points are from this line of best fit. The level of significance was set at P < 0.05.

## Results

### Analgesic effects of morphine in septic male rats

**Figure 3:**
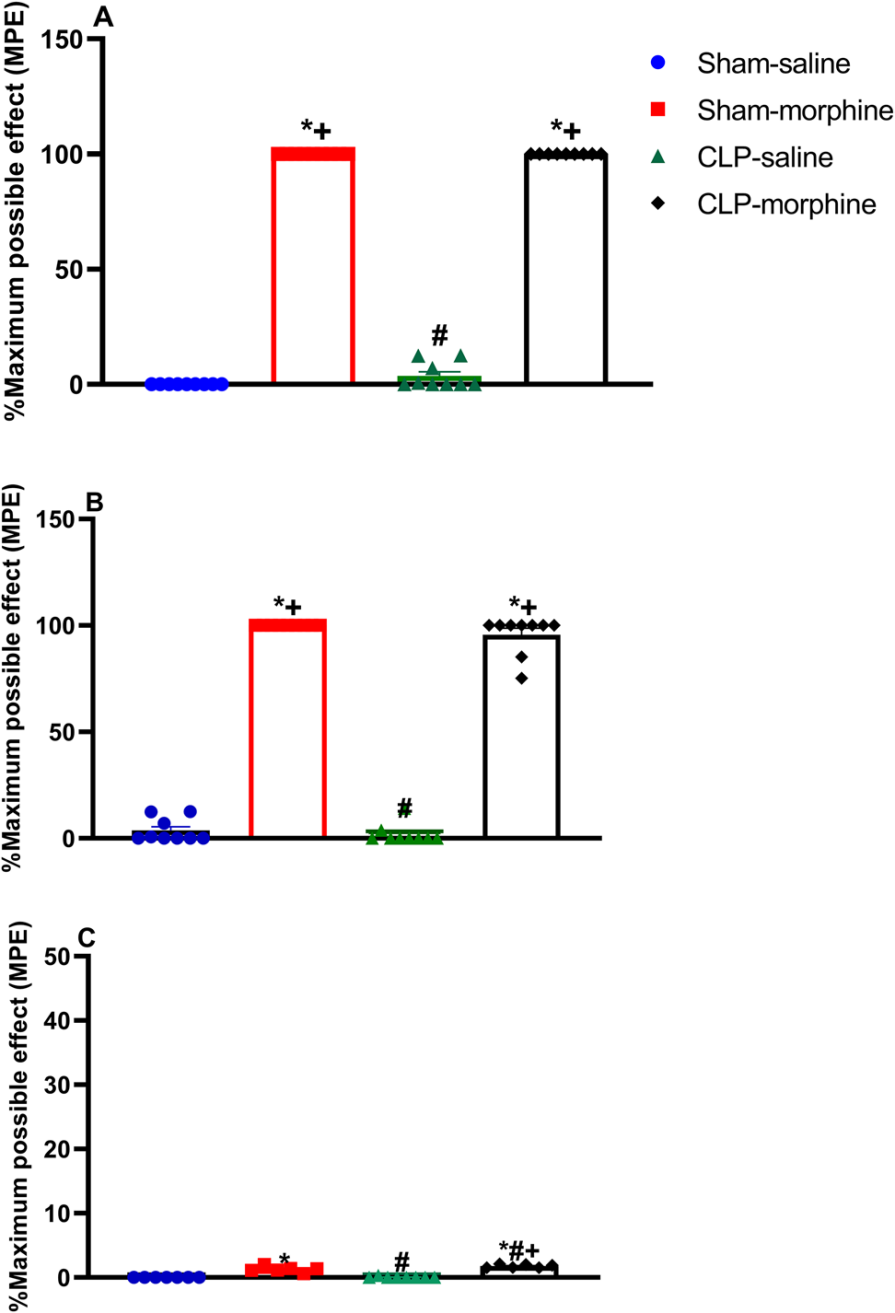
Effect of morphine (7 mg/kg, s.c.) on percentage maximum possible effect (%MPE)  (**A**) 24 h pre-surgery, (**B**) 6 h pre-surgery, and (**C**) 24 h post-surgery. Data were expressed as mean ± S.E.M (n = 6–9). One-way ANOVA followed by the Tukey’s post hoc test was used to measure statistical significance. *P < 0.05 vs. Sham-saline, ^+^P < 0.05 vs. CLP-saline, ^#^P < 0.05 vs. Sham-morphine.

Treatment of rats with morphine (7 mg/kg, s.c) at 24 h and 6 h before surgery caused a significant antinociceptive response expressed as % maximum possible effect (% MPE) compared to saline group, P < 0.05 (Fig. 3A,B). Morphine produced comparable effects both in sham and septic rats. The antinociceptive effect of morphine disappeared when measured 24 h after the last dose of morphine, i.e. 24 h post CLP or sham operation (Fig. 3C).

### Cardiovascular effects of morphine in septic male rats

**Figure 4:**
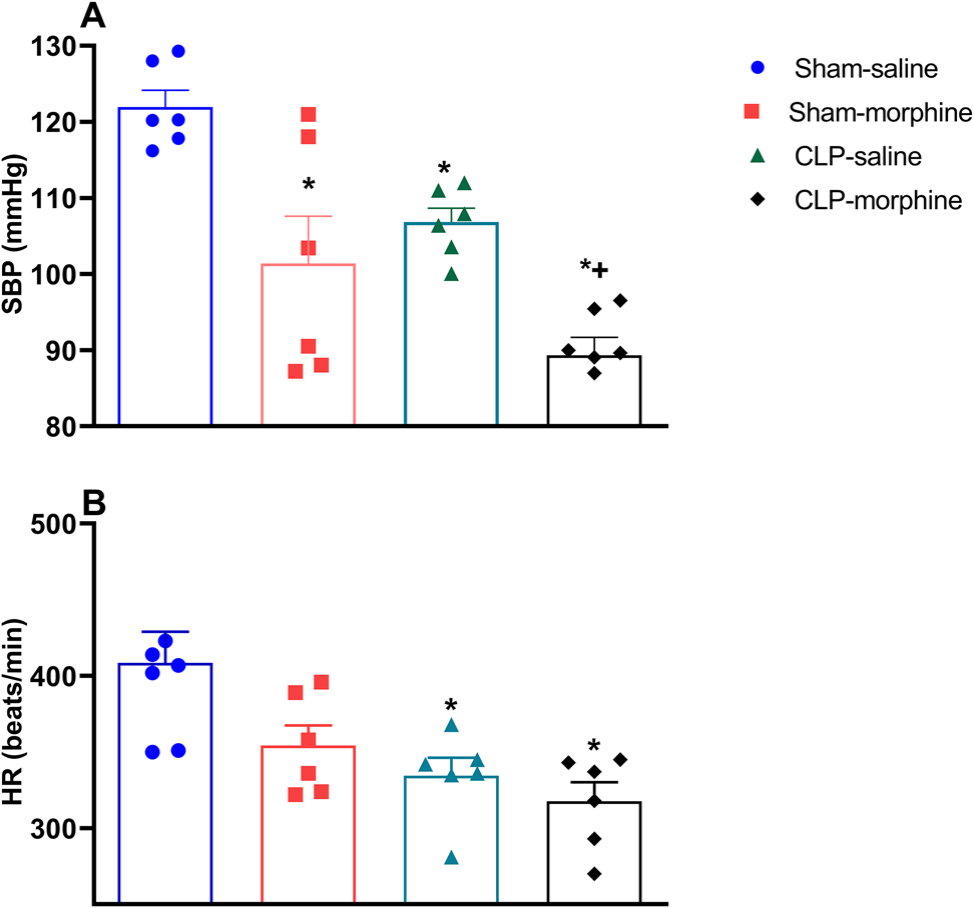
Effect of morphine (7 mg/kg, s.c.) on hemodynamic responses 24 h after CLP or sham operation. Data were expressed as mean ± S.E.M (n = 6–9). (**A**) Systolic blood pressure (SBP), (**B**) heart rate (HR), followed by the Tukey’s post hoc test was used to measure statistical significance. *P < 0.05 vs. Sham-saline, ^+^P < 0.05 vs. CLP-saline.

Cardiovascular measurement by the by tail-cuff technique were performed 24 h after CLP or sham operation. Figure 4 shows that CLP-induced sepsis elicited significant falls in SBP (Fig. 4A) and heart rate (HR)(Figure 4B) compared with sham rats. A similar fall in SBP was also observed in morphine-treated sham rats, whereas HR remained unaffected. Interestingly, morphine challenge of septic rats decreased SBP to levels that were signifacantly lower than those caused by individual insults (Fig. 4A).

### Effects of morphine on behavioral indices of Morris water maze (MWM) in septic male rats

**Figure 5:**
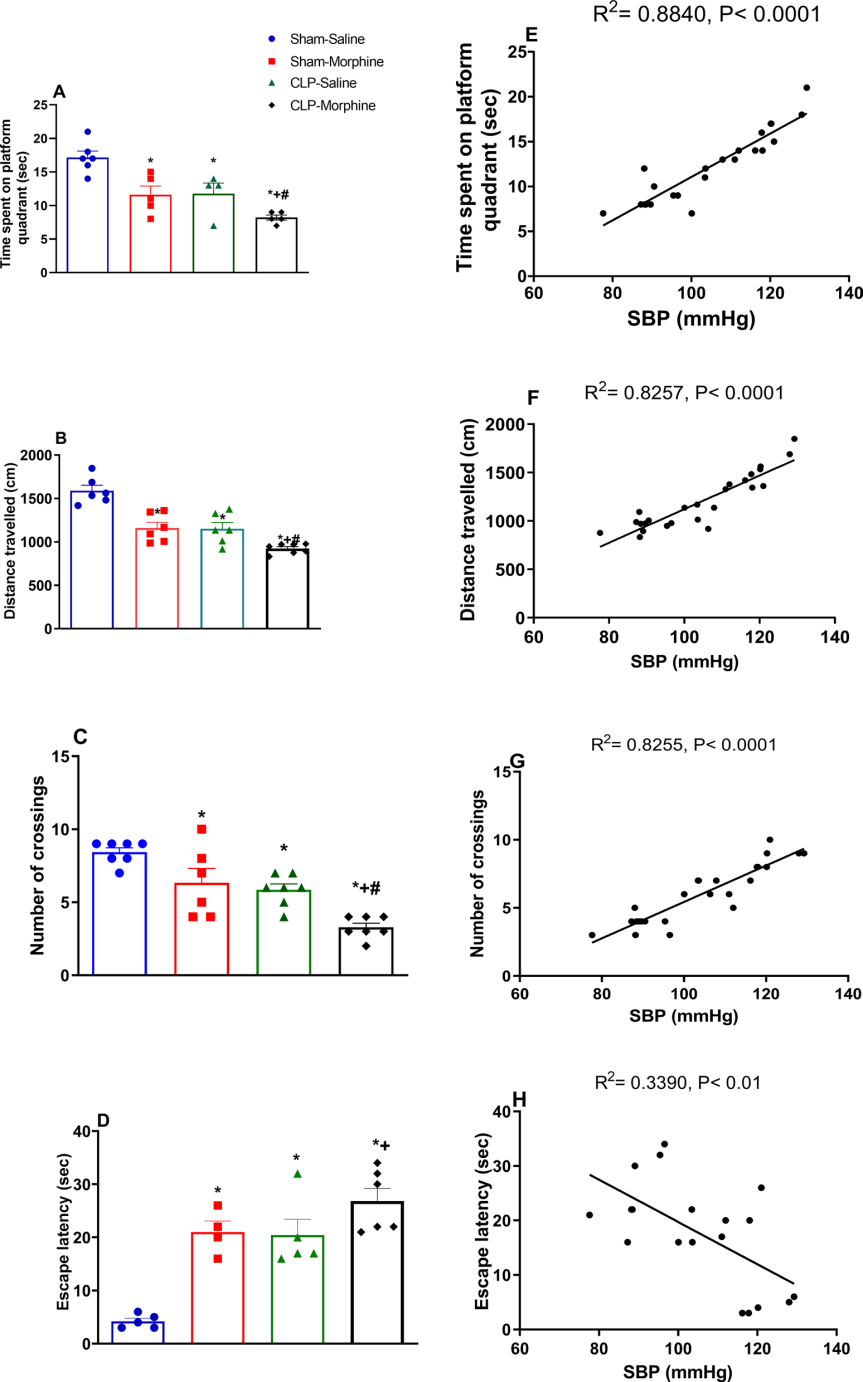
Behavioral and cognitive effects of morphine (7 mg/kg, s.c.) on CLP induced sepsis in rats using Morris Water Maze (**A**) time spent on platform quadrant, (**B**) distance travelled, (**C**) number of crossings, (**D**) escape latency, and the corresponding Pearson correlation with the systolic blood pressure, (**E**) time spent of platform quadrant, (**F**) distance travelled, (**G**) number of crossings, (**H**) escape latency. Data were expressed as mean ± S.E.M (n = 6–7). One-way ANOVA followed by the Tukey’s post hoc test was used to measure statistical significance. *P < 0.05 vs. Sham-saline, ^+^P < 0.05 vs. CLP-saline, ^#^P < 0.05 vs. Sham-morphine.

Figure 5 illustrates the effects of morphine and/or CLP on behavioral functions assessed by MWM. Compared with respective control values in sham rats, the treatment of sham rats with morphine or the induction of sepsis by CLP elicited similar increases in the escape latency time (Fig. 5D) and decreases in the time spent in the platform quadrant in the probe trial (Fig. 5A), the total distance travelled by rats (Fig. 5B), and the number of crossings to the platform quadrant (Fig. 5C). Further, the changes caused in these parameters in morphine-treated CLP rats were qualitatively similar but quantitatively greater than those caused by individual challenges (Fig. 5, P < 0.05).

The Pearson correlation analysis indicated a positive association between SBP (mmHg) and time spent on platform quadrant (Fig. 5E), distance travelled (Fig. 5F), number of crossings (Fig. 5G), (R^2^ = 0.8840, P < 0.0001, R^2^ = 0.8257, P < 0.0001, and R^2^ = 0.8255, P < 0.0001, respectively). On the other hand, the change in SBP was negatively correlated with escape latency (Fig. 5H) (R^2^ = 0.3390, P < 0.01).

### Effects of morphine on behavioral indices of the Y-maze test in septic male rats

**Figure 6:**
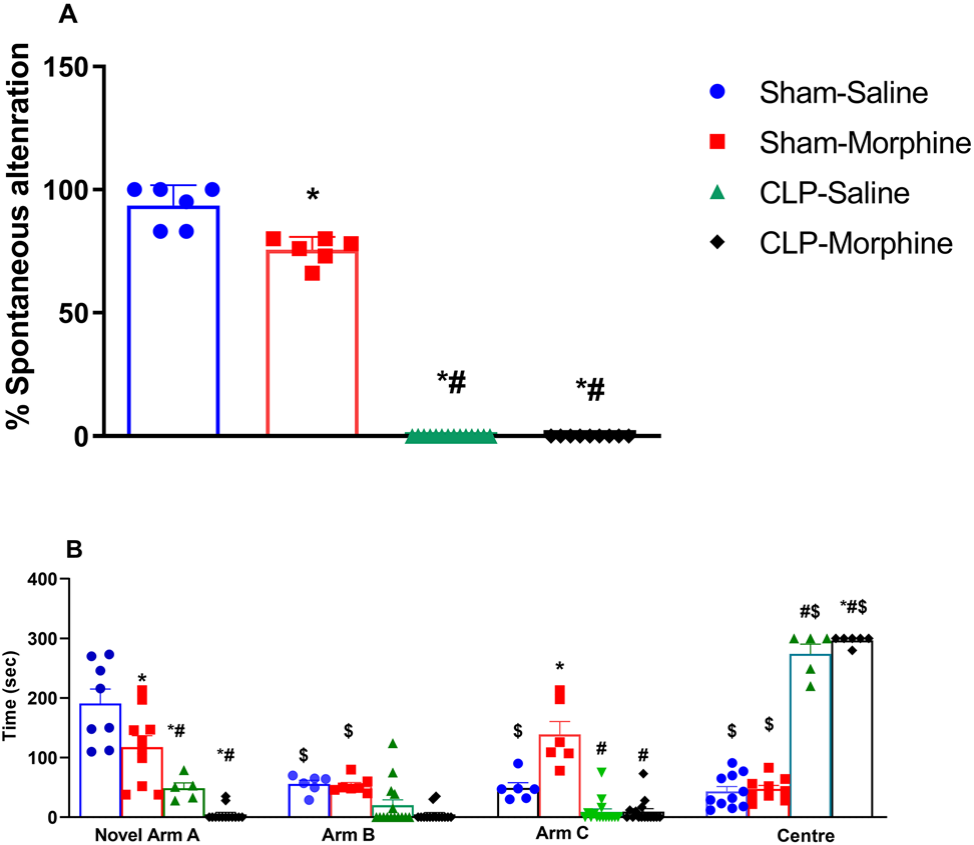
Effect of morphine (7 mg/kg, s.c.) on % spontaneous alternation and time spent in novel arm A, arms B and C and in the centre, of Y-maze in sham and CLP rats. The total duration of arm visits was recorded in the 5 min retention test. Data were expressed as mean ± S.E.M (n = 6–12). One-Way ANOVA followed by the Tukey’s post hoc test was employed to measure statistical significance. $ P<0.05 for difference in performance of rats in the novel arm vs. arms B & C and center of the same group. Difference in the performance between septic rats treated with morphine and other groups in the corresponding arm *P < 0.05 vs. Sham-saline, ^+^P < 0.05 vs. CLP-saline, ^#^P < 0.05 vs. Sham-morphine.

Morphine caused a significant reduction in the spontaneous alternation behavior compared to sham animals. The induction of sepsis by CLP virtually abolished the spontaneous alternation and this effect was maintained in morphine-challenged septic animals (Fig. 6A).

Either morphine or CLP significantly reduced the time spent on the novel arm by 38.46% and 74.40%, respectively, compared to sham group. Interestingly, morphine further decreased the time spent on the novel arm of septic rats (Fig. 6B). The CLP-morphine group spent 296.70 ± 3.33 s in the center and was significantly different from sham and sham/morphine groups. There was no significant difference in the time spent in the center between CLP animals and CLP-morphine-treated animals (Fig. 6B).

### Effects of morphine on behavioral indices of new object recognition (NOR) test in septic male rats

**Figure 7:**
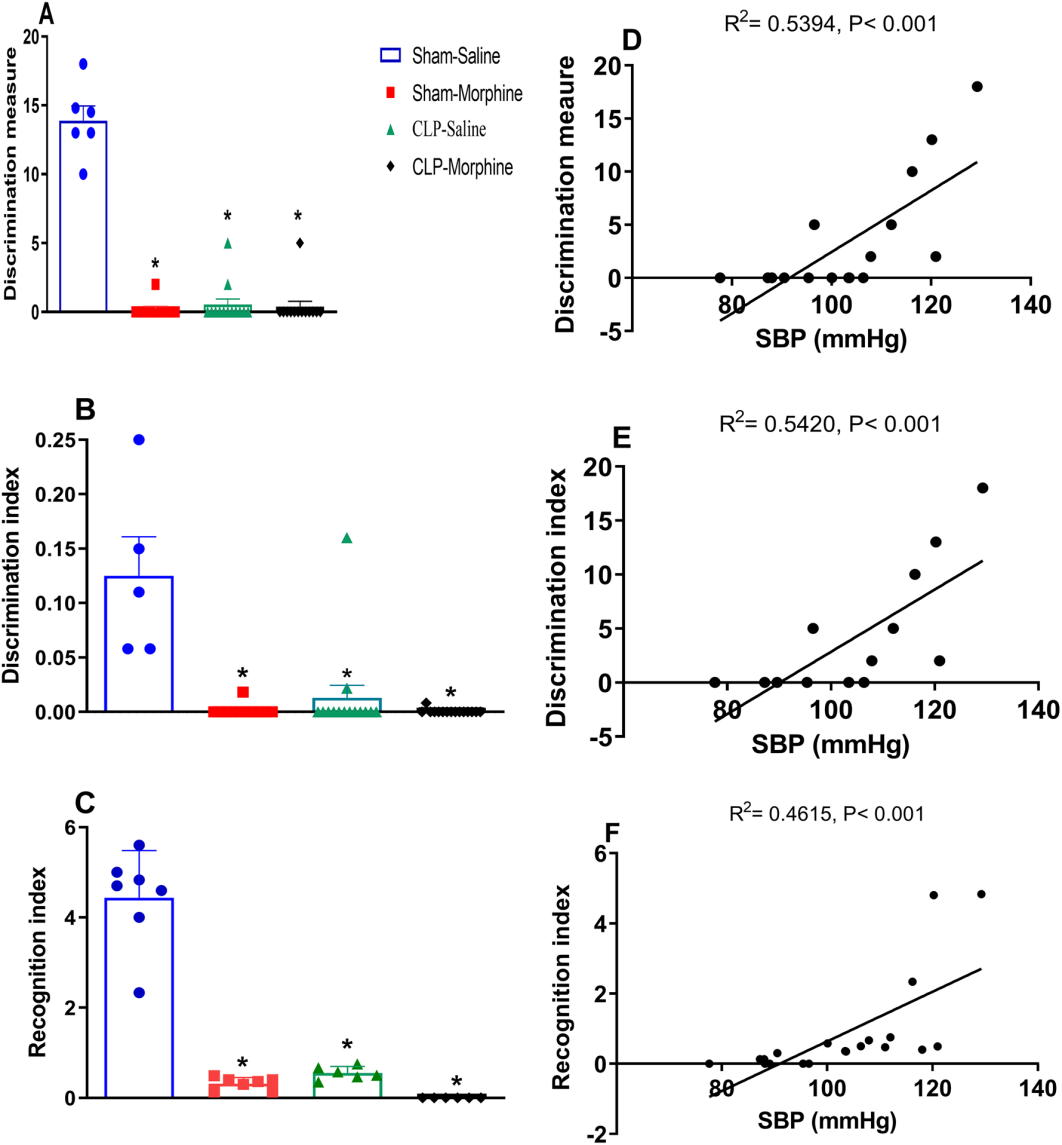
Effect of morphine (7 mg/kg, s.c.) on the behaviour of septic male rats in the New Object recognition test (**A**) discrimination measure, (**B**) discrimination index, (**C**) recognition index, and the corresponding Pearson correlation with systolic blood pressure, (**D**) discrimination measure, (**E**) discrimination index, (**F**) recognition index. Data were expressed as mean ± S.E.M. One-way ANOVA followed by the Tukey’s post hoc test was used to measure statistical significance. *P < 0.05 vs. Sham-saline.

All intervening challenges of morphine, CLP, and CLP/morphine were associated with similar and radical diminution of the discrimination measure (Fig. 7A), discrimination index (Fig. 7B) and recognition index (Fig. 7C) in the NOR test compared to sham rats. Pearson's correlation analysis showed a positive association between SBP (mmHg) and the discrimination measure (Fig. 7D) (R^2^ = 0.5394 P < 0.001), discrimination index (Fig. 7E) (R^2^ = 0.5420, P < 0.001) and the recognition index (Fig. 7F) (R^2^ = 0.4615, P < 0.001). The incredibly low scores approaching zero indicate a null preference in exploring the two objects implying that the time spent on novel object is equal to the time spent on familiar object.

**Figure 8:**
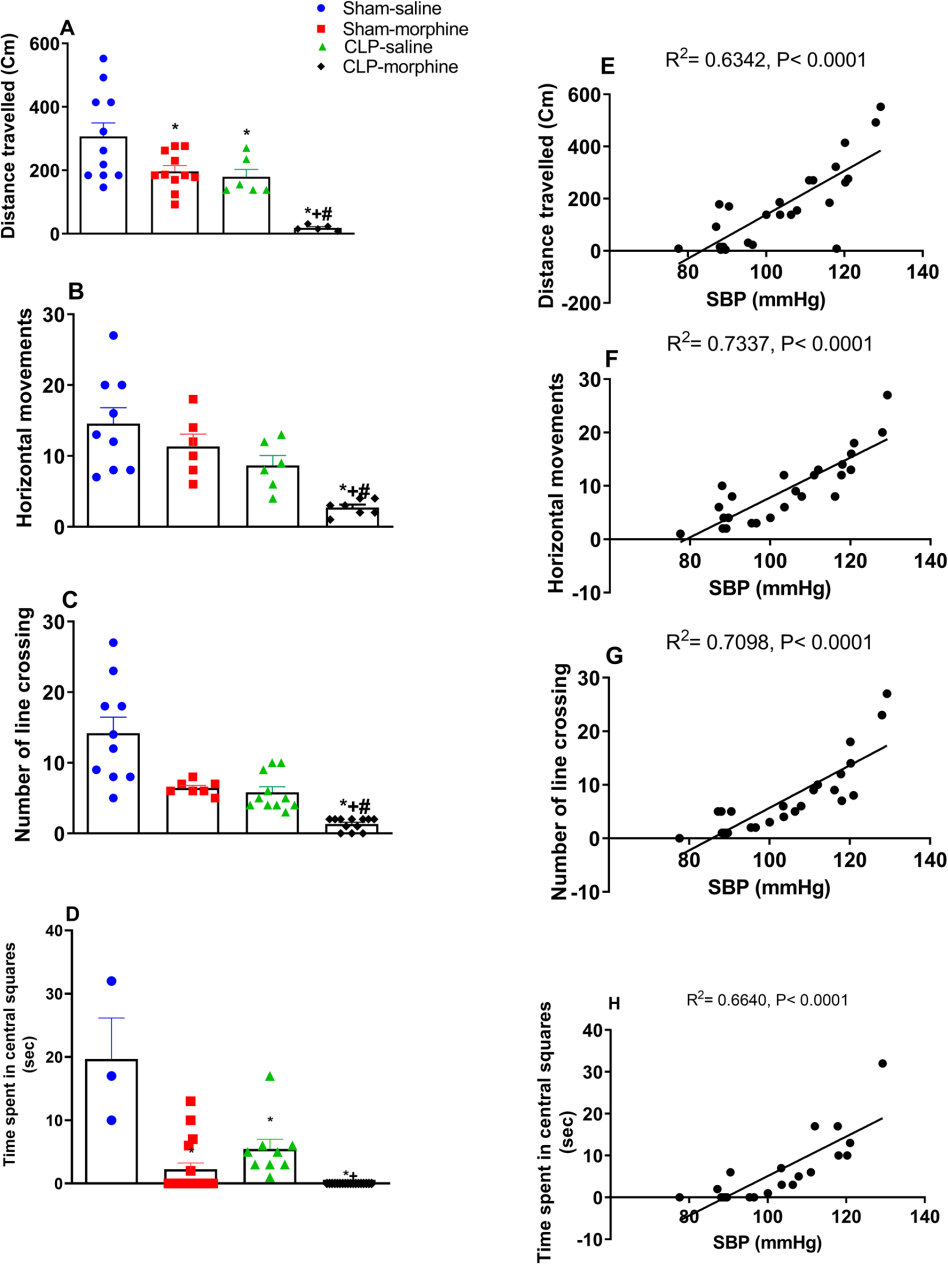
Effect of morphine (7 mg/kg, s.c.) on the exploratory functions in new object recognition test (**A**) distance travelled, (**B**) horizontal movements, (**C**) number of line crossings, (**D**) time spent in platform quadrant, and the corresponding Pearson correlation with the systolic blood pressure. (**E**) Distance travelled, (**F**) horizontal movements, (**G**) number of line crossing, (**H**) and time spent in central squares in sham and septic rats. Data were expressed as mean ± S.E.M (n = 6–11). One-way ANOVA followed by the Tukey’s post hoc test was used to measure statistical significance. *P < 0.05 vs Sham-saline, ^+^P < 0.05 vs. CLP-saline, ^#^P < 0.05 vs. Sham-morphine.

Figure 8 shows the effect of morphine and/or CLP on the distance travelled (Fig. 8A), horizontal movement (Fig. 8B), number of line crossings (Fig. 8C), and time spent in central squares (Fig. 8D) in the NOR test. Morphine treatment or induction of sepsis caused significant decreases in exploratory functions compared to sham animals. Furthermore, morphine treated septic rats exhibited the least exploratory behavior compared to the other three groups.

The use of morphine or the induction of sepsis by CLP significantly decreased the distance travelled in the open field (Fig. 8A), the number of horizontal movements (Fig. 8B) and the number of line crossings (Fig. 8C) compared to the sham group. Morphine further decreased these parameters in septic rats (Fig. 8A–C). Similarly, both morphine and CLP reduced the time rats spent in the central squares compared to sham rats (Fig. 8D). Interstingly, morphine treated septic rats did not spend anytime in the central squares, indicating that combined morphine/sepsis insult completely inhibited the animals exploratory behavior. Pearson correlation analysis indicated that SBP (mmHg) was positively associated with distance travelled (R2 = 0.6342, P < 0.0001 (A), horizontal movements (R2 = 0.7337, P < 0.0001 (B), number of line crossings R2 = 0.7098, P < 0.0001 (C), and time spent in central squares R2 = 0.6640, P < 0.0001 (D).

### Effect of morphine on serum IL-1β in septic male rats

**Figure 9:**
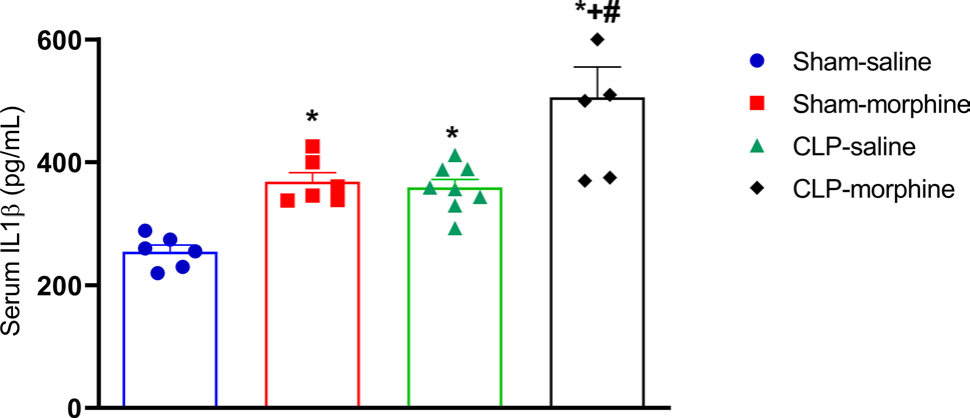
Effect of morphine (7 mg/kg, s.c.) on serum IL1β levels, in septic rats 24 h after CLP or sham operation. Data were expressed as mean ± S.E.M (n = 6–9). One-way ANOVA followed by the Tukey’s post hoc test was used to measure statistical significance. *P < 0.05 vs. Sham-saline, ^+^P < 0.05 vs. CLP-saline, ^#^P < 0.05 vs. Sham-morphine.

As shown in Fig. 9, the induction of sepsis by CLP or the treatment with morphine significantly increased the level of serum IL1β compared to saline treated rats (P < 0.05). Moreover, the treatment of septic rats with morphine caused more increases in serum IL1β to levels that were significantly higher than the effects of individual interventions (P < 0.05).

### Effects of morphine on hippocampal cellular morphology in septic male rats

**Figure 10:**
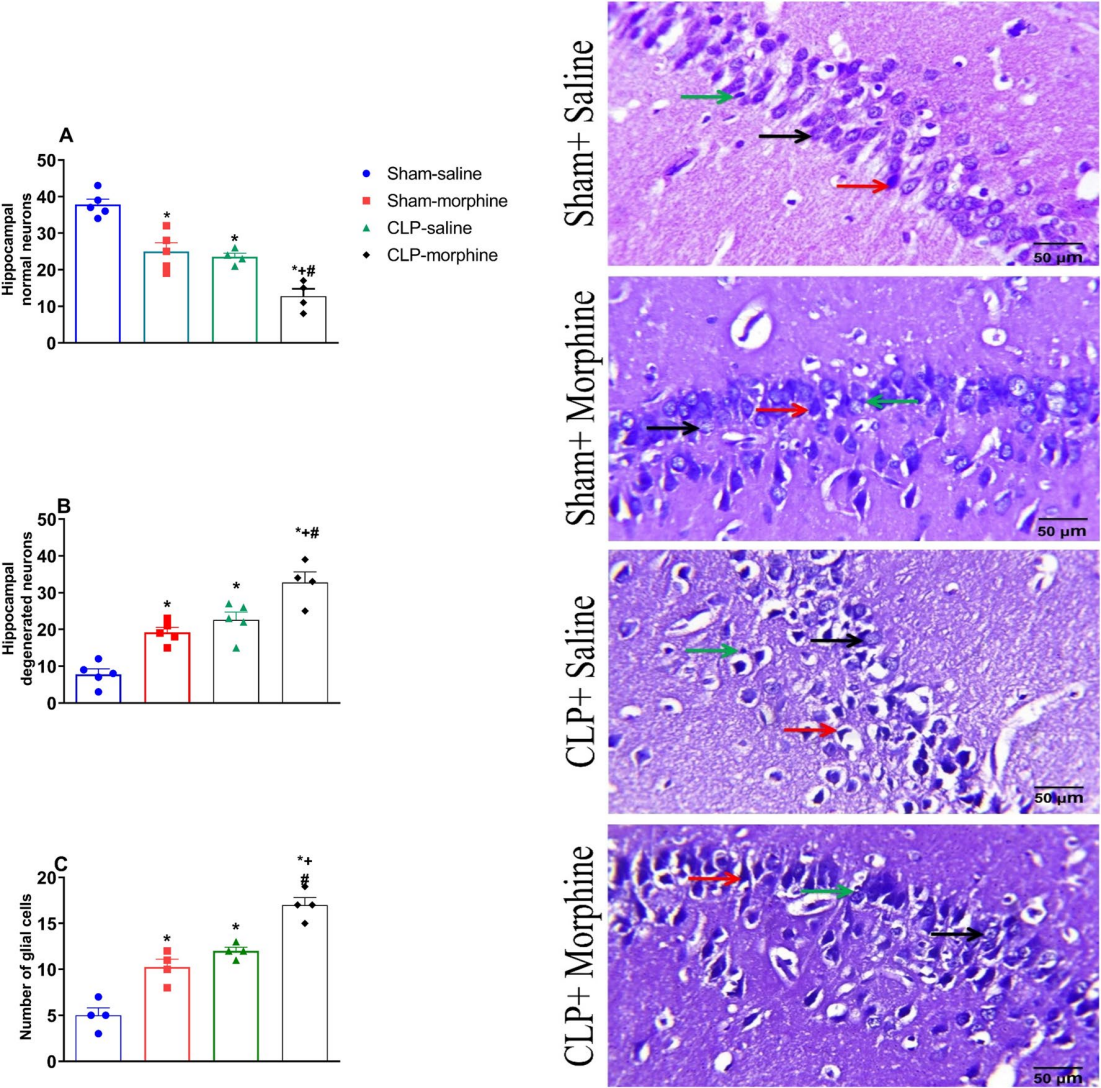
Effect of morphine (7 mg/kg, s.c.) on (**A**) normal neurons, (**B**) degenerated neurons, (**C**) glial cells in CA1 region of hippocampus of sham and septic rats. Representative photomicrographs of the rat hippocampus (CA1, HE, × 400) are shown. Data are means ± S.E.M. of observations. Normal neurons (black arrow), misaligned degenerated, shrunken neurons with pyknotic and hyperchromatic nuclei (red arrow) necrotic pyramidal with satellitosis and neuronophagia (green arrow). Data were expressed as mean ± S.E.M. One-way ANOVA followed by the Tukey’s post hoc test was used to measure statistical significance. *P < 0.05 vs. Sham-saline, ^+^P < 0.05 vs. CLP-saline, ^#^P < 0.05 vs. Sham-morphine.

As shown in Fig. 10, histological examination of the H&E stained CA1 area of the hippocampus tissues in sham rats demonstrated the faintly stained intact normal densely packed pyramidal neurons (black arrow). On the contrary, hippocampal tissues of rats treated with morphine and/or CLP showed distorted cellular morphology with increased pericellular space. The degenerated neurons are shrunken cells, misaligned, less cohesive with pyknotic, and hyperchromatic nuclei (red arrow) whereas the microglial cells are small and exhibit elongated nuclei with little cytoplasm Neuronal necrosis associated with satellitosis and neuronophagia was also noticed (green arrow). However, the most detrimental effects were observed in the CLP/morphine-treated group.

Figure 10 also depicts the differential hippocampal cell count of normal neurons (A), degenerated neurons (B) and glial cells (C). Compared to sham animals the number of normal neurons significantly decreased in morphine, CLP and CLP morphine treated animals (37.80 ± 1.53, 25.00 ± 2.35, 23.33 ± 1.45, 12.75 ± 2.02), respectively. On the other hand, sham rats treated with morphine, CLP and CLP morphine treated rats revelaled an increase in degenerated neurons compared to the sham group (19.20 ± 1.36, 22.60 ± 2.11, 32.75 ± 2.90, 7.80 ± 1.46) (P < 0.05). respectively. Likewise, the number of glial cells compared to sham group increased significantly in sham-morphine, CLP and CLP morphine treated rats (5.00 ± 0.82, 10.33 ± 1.20, 12.00 ± 0.58, 17.00 ± 1.16), respectively (Fig. 10).

### Effect of opioid receptor blockade by naloxone on morphine responses

**Figure 11:**
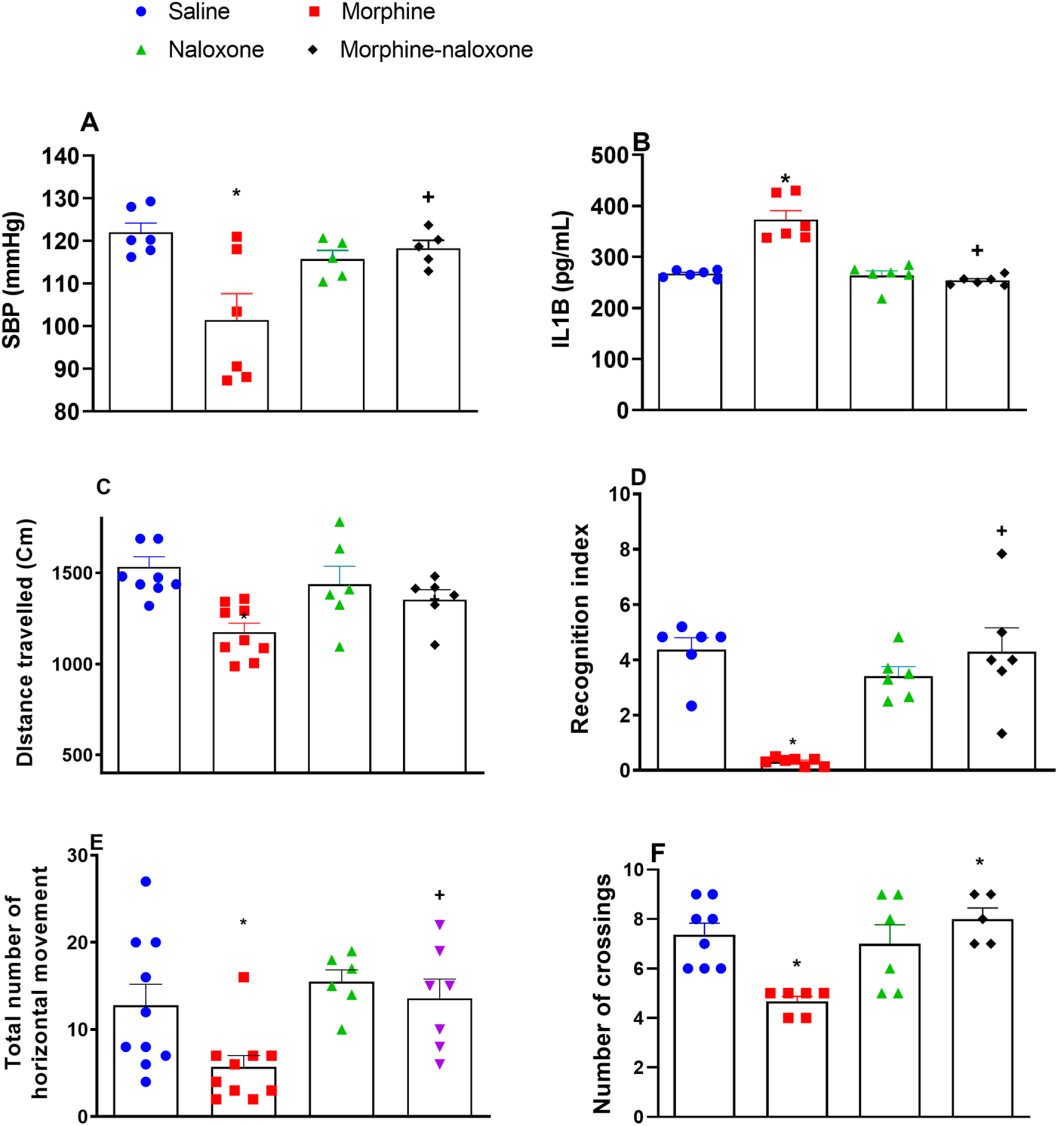
Effect of µ-opioid receptor blockade by naloxone (0.5 mg/kg, s.c.) on morphine (7 mg/kg, s.c.) evoked alternations in (**A**) SBP, (**B**) plasma IL1β, (**C**) distance travelled, (**D**) recognition index, (**E**) horizontal movements, (**F**) number of crossings recorded in the new object recognition test in sham rats. Data were expressed as mean ± S.E.M (n = 5–9). One-way ANOVA followed by the Tukey’s post hoc test was used to measure statistical significance *P < 0.05 vs. Saline, ^+^P < 0.05 vs. Morphine group.

To verify that the above described effects of morphine are mediated through its agonistic activity on the opioid receptor, we investigated the effect of µ-opioid receptor blockade by naloxone (0.5 mg/kg) on the morphine-evoked changes in SBP (Fig. 11A), serum IL-1β (Fig. 11B) and behavioral parameters (Fig. 11C–F) of the NORT in sham rats. Pretreatment with naloxone significantly reversed the morphine-evoked hypotension, inflammmation, and behavioral indicators measured in the NORT namely; recognition index (Fig. 11D), horizontal movements (Fig. 11E), and number of crossings (Fig. 11F). The sole treatment of sham rats with naloxone had no effect on the measured parameters (Fig. 11).

## Discussion

The current study reports several novel observations on the interaction of morphine with hemodynamic and behavioral responses induced by sepsis in rats. First, treatment of rats with morphine or the induction of sepsis by CLP caused hippocampal cellular defects, rises in serum IL-1β, and falls in blood pressure. The hypotensive response was associated and positively correlated with behavioral dysfunction that included disturbances in retention memory, acquisition, discrimination and exploratory functions. Second, the erupted hemodynamic, behavioral, and inflammatory imbalances were significantly exaggerated in rats exposed to the dual CLP/morphine insult. Third, the morphine effects in sham animals were substantially attenuated following the blockade of µ-opioid receptors by naloxone suggesting a prime functional role for these receptors in the evoked responses. Collectively, the data suggest that the simultaneous exposure to narcotic analgesics worsens the inflammatory response to the septic challenge and concomitant hemodynamic and behavioral sequels.

The current study presents the novel observation that treatment of septic rats with morphine caused exaggerated falls in SBP that exceeded the hypotensive response elicited by individual insults. The findings that single exposure to morphine or sepsis caused remarkable decreases in SBP are consistent with previous reports^65,66^. Vascular hypo-reactivity and systemic hypotension have been identified as key hallmarks of sepsis that follow the cytokine storm and overproduction of proinflammatory mediators like NF-κB, TNF-α, and interleukins^66,67^. The septic inflammatory response results in the generation of excessive quantities of nitric oxide and vasodilator prostanoids that impair vascular responsiveness and lower arterial pressure^13,68^. Alternatively, a hypotensive response to morphine has been reported after systemic^65^ and central administration^69^, but the underlying mechanism remains largely unidentified. However, a role for inflammation is likely as morphine has been shown to enhance the production of interleukin-12 and other pro-inflammatory cytokines in mouse peritoneal macrophages inflammation^70–73^ and to induce the translocation of gut gram-positive bacteria into the circulation^74^. Considering the inverse relationship between interleukins/iNOS availability and blood pressure control^66,67^, the inflammatory response initiated by morphine in the present study, as indexed by the concomitant rise in circulating IL-1β, could be a contributing factor to morphine hypotension. Likewise, the exaggerated elevations in circulating IL-1β might be responsible for the enormously dysregulated hemodynamics and substantial falls in blood pressure that followed the treatment of septic rats with morphine. Previous studies indicated that 24 h are required for morphine to sensitize the animals and increase the number of macrophages in peritoneal cavity^71^. Reportedly, morphine induced exacerbation of sepsis when injected 24 h before LPS administration^68^. Preemptive analgesia with intravenous morphine before surgical intervention, prevents central sensitization during surgery, reduces postoperative pain, analgesic requirements and is often used in clinical settings^75^.

Morphine interrupts brain circuits coupled with cognitive functions such as attention, learning and memory^38,76,77^. In the current study, several behavioral tests like the Y-Maze, Morris water maze and the new object recognition were employed to assess the behavioral influences of morphine and/or septic insults. Like the accentuated hypotensive and inflammatory responses, the data disclosed significantly greater losses in spatial memory, learning, and exploratory behavior in rats exposed to the joint CLP/morphine offence compared with individual ones. Compelling experimental evidence demonstrated impaired cognitive function in hypotension notably in the areas of attention, memory and processing speed in rotarod, cued learning and spatial learning tests^78^. Studies showed that individuals with low blood pressure may have inadequate control of cerebral blood flow in addition to inadequate blood flow adaptation to cognitive demands^79^.

More interestingly, the Pearson correlation studies revealed that the declines in blood pressure and behavioral functions were positively and significantly correlated with a maximum loss in behavioral performance seen in animals exhibiting the largest drop in SBP. Notably, it was found that high levels of TNF-α produced severe hypotension^80^ which in turn causes cognitive decline in behavioral tests as MWM^81,82^. Whether non-invasive measurement of blood pressure alone could predict outcomes on a cognitive task during training or Probe trails warrant investigation. Together, our findings establish a possible causal relationship between the amplified inflammatory response to the CLP/morphine insult and associated hemodynamic and behavioral traits.

One could argue whether the resulting cognitive impairment is attributed to treatment and sepsis insult or due to the limited ability of animals to perform the tasks. The confined position of animals in the center of the Y maze is due to deficit in exploratory behavior which is attributed to treatment and sepsis insult and not due to the sickness behavior of the animal. Another important factor which resulted in restricted animal movement to central position and almost abolished spontaneous alternation is attributed to increased anxiety. Sepsis causes 100% decrease in mice mobility in open field test post-sepsis^83^. Additionally, post-septic animals showed less overall exploratory behaviour in the novel object recognition task and increased anxiety-like behaviour in the elevated plus maze^84^. Both CLP and sham animals showed comparable drop-in activity in the 4 h post-surgery monitoring period presumably due to anesthesia, laparotomy and mobilization of abdominal contents. No significant difference between sham operated and unoperated animals in the locomotor behavior was detected 2–12 h post-surgery^85^. That said, surgery as such does not appear to affect locomotor behavior and the decrease in CLP rats’ activity relates to sepsis progression. Moreover, cognition impairment is not only judged by declined locomotion parameters but was reinforced by the observed neuroinflammation and hippocampal injury.

Histological examination revealed a distorted cellular morphology in hippocampal tissues excised from CLP/morphine rats that surpassed respective changes in rats with single insults. The CLP/morphine challenge caused more pronounced neuronal necrosis together with bigger decrements and increments in the number of normal neurons and glial cells, respectively. Further, these changes were noted in the CA1 region of the hippocampus, a key neuroanatomical area that arbitrates behavioral responses elicited by morphine^86^ as well as sepsis^87^.

Sepsis-related cognitive decline is reported in rats after recovery from the abdominal infection after the CLP surgery^88^. Therefore, it is likely that the subsequent behavioral changes in the current study are indeed related to sepsis induced cognitive decline. Only rats with adequate health status and locomotor activity were included in the current study to rule out the possibility that the behavioral and/or cognitive aberrations are related to the acute CLP-infection itself. Additionally, matched weight, age and sex sham operated groups are included in all experiments.

The elevation in circulating IL-1β is followed by a rise in the central expression of IL-1β and other inflammatory mediators such as TNF-α and COX-2^89^, thus, it is conceivable to implicate the exacerbated levels of blood IL-1β in hippocampal tissue damage and behavioral defects induced by the combined CLP/morphine insults. Opioids induce a pro-inflammatory response within the CNS, primarily via the microglia, the predominant immunocompetent cell within the CNS^90^. Morphine affects glia by binding to the innate immune TLR4, leading to the release of proinflammatory cytokines, opposition of morphine analgesia and further prolonging postsurgical cognitive dysfunction^16,26^. Morphine causes priming of microglia within different central loci including hippocampus, the spinal cord as well as the trigeminal nucleus caudalis. When these primed microglia are shortly challenged with a second immune insult, the pro-inflammatory response is exacerbated^91^. Recently demonstrated, morphine was found to induce long-lasting upregulation of the endogenous TLR4 ligand, high mobility group box 1 (HMGB1) within rat hippocampus, causing prolonged neuroinflammation and synaptic dysregulation^16^. On the other hand, TLR4 are expressed on a range of peripheral immune cells. Activation of TLR4 by morphine elicits a neuroinflammatory state^90^. Morphine relates to peripheral nerve injury, that induces neuropathic pain, via TLR4 signaling^90,92^. Activated peripheral immune cells can translocate to the CNS^93^. That said, the sensitizing effects of systemic morphine, in the current study, may not be restricted to the central glial cells.

It is imperative to comment on two features of the behavioral response to morphine. First, the morphine effects were observed 30 h after the last dose of the drug (i.e., 24 h after CLP), a time at which the antinociceptive action was no longer noticeable. This fits well with the presumption that no timely link exists between the antinociceptive action of morphine on the one hand, and the cognitive depressant and neuroinflammatory actions on the other^94^. The lack of morphine analgesia when measured 24 h post-CLP may be accounted for by the tendency of the septic insult to induce a state of hyperalgesia or allodynia^95^. The second feature relates to the abolition of morphine responses in sham animals upon pre-treatment with the µ-opioid receptor blocker naloxone (Fig. 11), suggesting the importance of the µ-opioid receptors in the elicitation of behavioral effects of the drug as documented by other^38,73,90,96–99^. It is worth mentioning that the results of the current study established the deleterious impact of morphine on hemodynamic and behavioral responses in normal animals which prompted us to unveil the possible mechanism (s) underlying these changes. Morphine effects were substantially attenuated following the blockade of µ-opioid receptors by naloxone suggesting a detrimental role for these receptors in the evoked responses. Morphine increased the production of IL-12 and other proinflammatory cytokines is blocked by naloxone^72^. Indeed, pharmacologic and in silico docking studies suggest a basic role for opioid receptors in the hypotensive and inflammatory actions of narcotic opioids^100–102^.

In summary, this experimental study illustrates that morphine elicits correlative exacerbations of hemodynamic and behavioral irregularities induced by sepsis. These defects appear to be related to the inflammatory and hippocampal cellular aberrations induced by the CLP/morphine challenge. The clinical relevance of the present findings is warranted especially in intensive care units where sepsis is the most prevalent cause of death and opioids are the analgesics of choice for pain control.

### Supplementary Information

Supplementary Information.

## Data Availability

Raw data are provided as an additional Supporting File.
