# Supplementary material for: Morphine aggravates inflammatory, behavioral, and hippocampal structural deficits in septic rats
Source: Sci Rep. 2023 Dec 5;13:21460. doi: 10.1038/s41598-023-46427-y (PMC10697987; doi:10.1038/s41598-023-46427-y)
Supplement: Supplementary file 1 — Supplementary Information. [file 41598_2023_46427_MOESM1_ESM.docx]

**EXPERIMENTAL RESULTS ON COGNITION OF A CONTROL RAT**

**Experiment No. 1**

Time = 9mins

Number of Arm Entries = 10

Entries = BCABCBABCA

Spontaneous Alterations= BCA, CAB, ABC, CBA, ABC, BCA = 6

Spontaneous Alterations % = # spontaneous alterations x 100%

Total number of arm entries – 2

= 6 x 100% = **75%**

10-2

**Experiment No. 2**

Time = 9 mins 23 secs

Number of Arm Entries = 11

Entries = BACBACBACBA

Spontaneous Alterations= BAC, ACB, CBA, BAC, ACB, CBA, BAC, ACB, CBA. = 9

Spontaneous Alterations % = # spontaneous alterations x 100%

Total number of arm entries – 2

= 9 x 100% = **100%**

11-2

NB: The lower the % spontaneous alteration the more the cognitive impairment.

Experiment CONDUCTED ON 1^ST^ DEC 2019

|  | % Spatial Acquisition (Y-Maze) | Discrimination measure (d1) New Object recognition | Discriminative Index (d2) New Object recognition | Recognition Index (d3) New Object Recognition | Morris Water Maze (no. of crossings |
| --- | --- | --- | --- | --- | --- |
| Rat 1 | 71.43 | 22secs | 0.037 | 6.33 | 4 |
| Rat 2 | Moved three times | 6secs | 0.01 | 11.17 | 5 |
| Rat 3 | 75.00 | 4secs | 0.007 | 1.67 | 6 |
| Rat 4 | 71.43 | 44secs | 0.073 | 10.33 | 6 |
| Rat 5 | 100.00 | -5secs | -0.008 | 0.17 | 5 |

**COLLECTIVE TABLE FOR ALL TRIALS**

| **Y-Maze** | | | | | | **Lactate mmol/L** | | **BP** | | | | | | **Trial No/ Date** | |
| --- | --- | --- | --- | --- | --- | --- | --- | --- | --- | --- | --- | --- | --- | --- | --- |
| **CLP** | | | **Sham** | | | **CLP** | **Sham** | **CLP** | | | **Sham** | | |  |  |
| **48hrs** | **24hrs** | **0hrs** | **48hrs** | **24hrs** | **0hrs** | **24hrs** | **24hrs** | **24hrs** | **6hrs** | **0hrs** | **24hrs** | **6hrs** | **0hrs** |  |  |
|  |  |  |  |  |  |  |  | 1)Dead | 1)74  2)Dead  3)Dead | 1)107  2)103  3)111 | 1)118  2)101 | 1)85  2)85 | 1)123  2)112 | **1)17.09.19** |  |
|  |  |  |  |  |  |  |  | 1)65  2)Dead  3)75  4)138  5)Dead |  |  |  |  |  | **2)24.09.19** |  |
|  |  |  |  |  |  |  |  | 1)140  3)113  4)110  5)128  6)112 |  | 1)130  3)142  4)148  5)132  6)151 | 2)Dead |  | 2)130 | **3)07.10.19** |  |
| - | 3)91% | 2)67% | - | All did not move | 1)37.5%  2)77.8% | 1)19.42  2)5.01  3)4.45 | 1)2.56  2)2.81  3)2.51 | 1)135.3  2)90.2  3)Dead |  | 1)122.7  2)115.5  3)112.7 | 1)120.2  2)125.8  3)125.6 |  | 1)122.6  2)118.3  3)118 | **4)16.10.19** |  |
| 2)40%  3)No movements  4)Dead  5)50%  6)Dead | All did not move, one dead before Y-maze test | 2)36.3%  3)100%  4)75%  5)72.7%  6)40% | 1)66.7% |  | 1)50% | 2)1.83  3)5.03  4)6.62  5)2.64  6)7.08 | 1)2.60 | 2)114  3)100.7  4)99 Dead  5)113.8  6)Dead |  | 2)112.5  3)115.7  4)113.3  5)120.9  6)110.9 | 1)117.6 |  | 1)110.5 | **5)29.10.19** |  |

NB: No Y maze was conducted in trial 1,2 and 3. In trail 4 all animals were not adequately trained and only Sham 1, 2 and CLP 2 moved at 0hrs and all animals did not move at 24hrs except CLP 3. No Y maze was conducted at 48hrs on trial 4. In trial 5 CLP 2,3 and 4 received 2 punctures while CLP 5 and 6 received 4 Punctures.

**Calculations for Y maze**

**Formula:**

**Spontaneous Alterations % = Number of Spontaneous Alterations x 100**

**Total no. of Entries - 2**

**Trial no 4**

Sham 1 0hrs

Time=8mins

No. of Entries= 10 CBCBABABCA

Spontaneous Alterations (SA)=3

%SA= (3/10-2) x100= **37.5%**

Sham 2 0hrs

Time=8mins

No. of Entries=11 BACBACBABCA

SA=7

%SA= (7/11-2) x 100=**77.8%**

CLP 2 0hrs

Time=8mins

No. of Entries=5 CBABC

SA=2

%SA = (2/5-2) x 100=**67%**

CLP 3 24hrs

Time=8mins

No. of Entries=13 BACBCABCABCAB

SA=10

%SA= (10/13-2) x 100 = **91%**

**Trial 5**

Sham 1 0hrs

Time=8mins

No. of Entries=4 BCAC

SA=1

%SA= (1/4-2) x 100 = **50%**

Sham 1 48hrs

Time=8mins

No. of Entries=5 BABCA

SA=2

%SA= (2/5-2) x 100 = **67.7%**

CLP 2 0hrs

Time=8mins

No. of Entries=13 ABCBCACACBCAC

SA=4

%SA= (4/13-2) x 100 = **36.3%**

CLP 2 48hrs

Time=8mins

No. of Entries=7 CBCACBC

SA=2

%SA= (2/7-2) x 100 = **40%**

CLP 3 0hrs

Time=8mins

No. of Entries=7 CABCABC

SA=5

%SA= (5/7-2) x 100 = **100%**

CLP 4 0hrs

Time=8mis

No. of Entries=6 CBACBC

SA=3

%SA= (3/6-2) x 100 = **75%**

CLP 5 0hrs

Time=8mins

No. of Entries=13 BCABCACABCBAC

SA=8

%SA= (8/13-2) x 100 = **72.7%**

CLP 5 48hrs

Time=8mins

No. of Entries=8 BCACABCB

SA=3

%SA= (3/8-2) x 100 = **50%**

CLP 6 0hrs

Time=8mins

No. of Entries=7 CBCBACA

SA=2

%SA= (2/7-2) x 100 = **50%**

**Cecal ligation and puncture procedure**

**
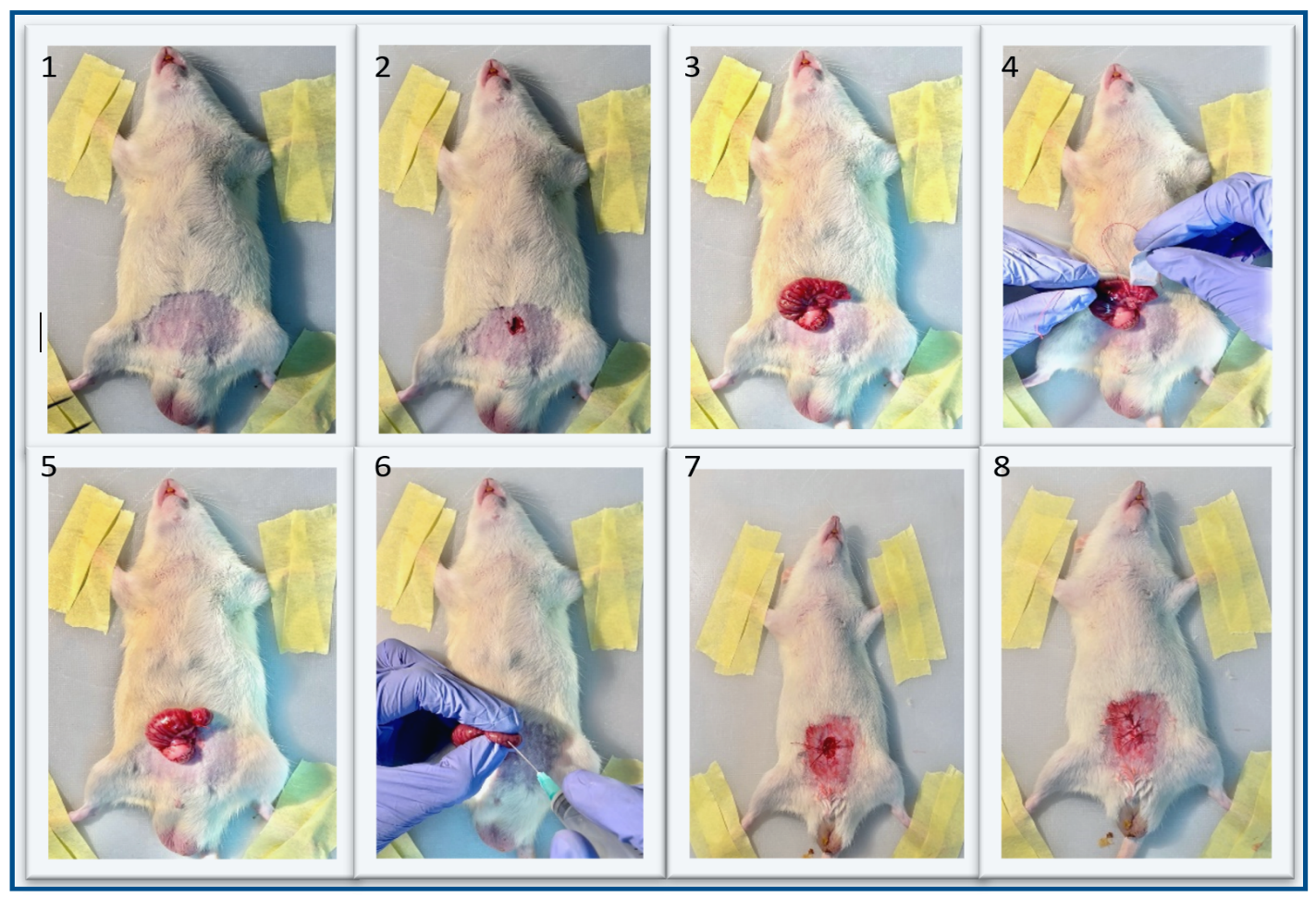
**
